# Oligosaccharides and diet-related dilated cardiomyopathy in beagles

**DOI:** 10.3389/fvets.2023.1183301

**Published:** 2023-07-24

**Authors:** Elise Bokshowan, T. Dylan Olver, Matheus de O. Costa, Lynn P. Weber

**Affiliations:** ^1^Department of Veterinary Biomedical Sciences, University of Saskatchewan, Saskatoon, SK, Canada; ^2^Department of Large Animal Clinical Sciences, University of Saskatchewan, Saskatoon, SK, Canada

**Keywords:** dilated cardiomyopathy, grain-free dog food, oligosaccharides, dietary fiber, taurine

## Abstract

**Introduction:**

In 2018 the US Food and Drug Administration reported a potential link between grain-free, legume-containing dog foods and the development of canine dilated cardiomyopathy in atypical breeds. One hypothesis was that high oligosaccharide content in legumes reduced bioavailability of taurine, an amino acid with some previous links to canine dilated cardiomyopathy.

**Methods:**

To address this hypothesis, in the present study, 8 Beagle dogs consumed four diets: a husbandry commercial dental diet, and three test diets formulated with either 30% rice (control), 30% pea (grain-free) or 30% rice with the addition of 1% raffinose (the predominant oligosaccharide found in peas). The study was conducted in a randomized, crossover design with 5 week feeding periods. Measurement of basic health parameters (weight, body condition score, complete blood cell count, chemistry panel), plasma amino acids, cardiac biomarkers (plasma N-terminal pro-brain natriuretic peptide (NT-proBNP) and cardiac-specific troponin I), fecal bile acids and echocardiographic exams were completed pre-study after feeding the husbandry diet as well as after each test feeding period.

**Results:**

Echocardiography showed 9–11% reduction in ejection fraction and 17– 20% greater left ventricular end systolic volume with the husbandry diet compared to both grain-containing test diets. Concentrations of plasma NT-proBNP were 1.3–2 times greater after the husbandry diet compared to the grain-based diet, with the oligosaccharide and pea-based diets showing intermediate levels. Plasma taurine levels were unchanged across diets, while plasma methionine levels were highest and cysteine/cystine levels were lowest after dogs ate the husbandry diet.

**Discussion:**

Results indicate that raffinose in the diet is sufficient, but not required to see an increase NT-proBNP, but did not induce any changes in cardiac function after 5 weeks of feeding. Whether this could progress to reduction in cardiac function with longer term feeding is uncertain. A reduced cardiac function along with the greatest increase in NT-proBNP was observed after feeding the husbandry diet that contained the highest amount of insoluble fiber but did not contain legumes or oligosaccharide. Further research into the impact of insoluble fiber in the dental diet is needed to support these novel observations.

## 1. Introduction

In 2018, the US Food and Drug Administration (FDA) reported they were investigating the increased incidence of canine dilated cardiomyopathy (DCM) in breeds not predisposed to the disease who were being fed grain-free dog food with high legume or potato content (1). The FDA provided updates on the investigation as new information was available, but in none of the updates have they been able to provide an explanation for the observed link (2–5). Since the initial report, research into possible mechanisms has been ongoing with many causes proposed. The causal link however, is yet to be established, and in December 2022 the FDA announced they would no longer be providing updates until critical scientific discoveries are made and urged more research (6).

Dilated cardiomyopathy is a serious chronic myocardial condition characterized by impaired systolic function, left ventricle chamber dilation and ventricular wall thinning that can be fatal (7). Diagnosis of DCM is ideally done through echocardiography and heart failure from DCM progression is defined as an ejection fraction (EF) < 40% (7). Additional screening for DCM includes monitoring for elevations in either a marker of cardiac stretch, blood levels of N-terminal pro-brain natriuretic peptide (NT-proBNP), or a marker of cardiomyocyte damage, cardiac-specific troponin I (8–10). Previously, genetic DCM was known to occur in certain breeds with a predisposition including Deerhounds, Newfoundlands, Irish Wolfhounds, Doberman Pinschers and Cocker Spaniels (11). However, with the FDA report, a nutritionally-mediated DCM has become apparent with peas being the most common ingredient among the reported DCM cases (12). Since then, DCM has been reported to be nutritionally inducible in Golden Retrievers or a mixed breed population after feeding diets with high pea content (13–15), while another study failed to find any echocardiographic changes despite increased blood markers of cardiac disease (16). Moreover, switching dogs to a grain-inclusive or pulse-exclusive diet has been reported to improve cardiac function in dogs with diagnosed DCM (8, 17–19).

Initial research surrounding diet-related DCM centered on the sulfur containing amino acid taurine, whose deficiency has an established link to some forms of canine DCM (20–22). Taurine is found exclusively in animal protein and is completely absent from plant sourced proteins. However, taurine is not considered an essential amino acid for dogs because they can synthesize it from the other sulfur-containing amino acids, methionine, and cysteine (23). Taurine has many roles in the body and is found in particularly high concentrations in heart muscle, where it is thought to be important for cellular energy utilization and membrane integrity (24). Another important function is in the formation of the bile acid taurocholate by conjugating taurine with cholesterol-derived bile acids (25). Taurocholic acid (TCA) is the only conjugated bile acid synthesized by dogs that is then released into the small intestine to assist with the absorption of dietary fat. Most bile acids are reabsorbed and recycled by the body, while some is lost in the feces, which must then be replaced with *de novo* synthesis of TCA (26). There is also the potential for TCA to be transformed by intestinal bacteria through deconjugation of taurine (27). This results in the loss of taurine through consumption by the microbiome, and the formation of secondary bile acids, the majority of which are excreted in the feces (28). Compared to grains, pulses have relatively high concentrations of soluble fiber and oligosaccharides, which have the potential to bind with bile acids in the intestine and facilitate their excretion in stool (28–31). Moreover, oligosaccharides are a direct source of nutrition to the microbiome, and it may be that a higher oligosaccharide intake supports a larger microbial population, leading to reduced bile acid reabsorption and an increase loss in taurine to microbial metabolism (32, 33).

Thus, the connection between grain-free diets and canine DCM may be related to the high oligosaccharide content of legumes, which reduces taurine availability through microbial catabolism and increased fecal bile acid loss. Therefore, it was hypothesized that the consumption of oligosaccharide-containing diets may cause DCM-like cardiac changes and increase fecal bile acid losses, irrespective of the presence or absence of legumes or grains. To address this hypothesis, eight adult beagles were fed three high protein test diets in a randomized, crossover design of 5 week feeding periods. The test diets included a control rice-based diet (GB), an oligosaccharide-enriched rice-based diet (Oligo), and a grain-free, pea-based diet (GF). At the start of the study and at the end of each feeding period, cardiovascular function was assessed using echocardiography as well as plasma NT-proBNP and troponin measured. At the end of each feeding period, measurements included standard blood chemistry and cell counts, plasma levels of sulfur-containing amino acids (taurine, methionine and cysteine/cystine), fecal total bile acid and fecal TCA.

## 2. Materials and methods

All procedures were conducted following a protocol approved by the University of Saskatchewan’s Animal Research Ethics Board and adhered to the Canadian Council on Animal Care guidelines for humane animal use (protocol #20190055). Chemicals and ingredients were obtained from Sigma Aldrich (Markam, ON, Canada) unless specified otherwise.

### 2.1. Animals

Eight adult colony Beagle dogs (4 spayed females, 4 neutered males, ages 3–5 years old) were obtained from certified scientific breeders (Marshal Bioresources, North Rose, NY, USA and King Fisher International, Stouffville, ON, Canada). The dogs were housed at the Western College of Veterinary Medicine’s Animal Care Unit (ACU) at the University of Saskatchewan. During the day, the animals were group housed in a large enclosure with toy enrichment, but individually kenneled overnight and while feeding. Except in inclement weather, the dogs had 24-h access to outdoor runs. Food was provided individually twice daily, with water available *ad libitum*. Dogs received daily walks and socialization. Health was monitored daily, with body weights collected weekly and teeth brushing using only water daily. A veterinarian examined the dogs prior to the start and after the end of the study to ensure good health, and all vaccinations and deworming kept up to date.

### 2.2. Diets

Diets were formulated using Concept 5 software (Creative Formulation Concepts, Pierz, MN, USA) to meet the Association of American Feed Control Officials (AAFCO) (34) nutrient requirements for adult dogs, with 30% inclusion of either rice or pea flour (Table 1). Three test diets were formulated for the feeding trials, including a control, grain-based rice diet (GB), the rice diet with a 1% inclusion of raffinose, the primary oligosaccharide found in peas (Oligo), and a grain-free, pea-based diet (GF) with a 30% inclusion of whole smooth pea flour (CDC Inca) (35). A previous study found that oligosaccharide content of peas ranged from 2 to 6% (dry matter or DM), which guided the decision to add 1% raffinose to approximate the naturally occurring oligosaccharide content of the GF diet with 30% inclusion of pea flour (12). Celite^®^, a non-digestible marker made of diatomite, was included at 1% for the purpose of measuring total tract apparent digestibility. In order for the test diets to maintain similar nitrogen content, pork meal content was reduced in the GF diet due to the higher protein content of the pea flour compared to the rice flour. Moreover, potato flour was used to balance diets, with potato flour replacing a portion of the pork and bone meal in the GF diet compared to the GB diet.

**Table 1:** Formulation of test diets.

Ingredients (% inclusion as fed)	GB	Oligo	GF
Rice flour, white	30	30	–
Pea flour, CDC inca	–	–	30
Pork and bone meal	30	30	20.6
Salmon meal	10	10	10
Potato flour	18.6	17.6	28
Canola oil	6	6	6
Salmon oil	3	3	3
Raffinose	-	1	-
Celite	1	1	1
Vitamin/mineral premix	1	1	1
Salt	0.3	0.3	0.3
Choline chloride	0.1	0.1	0.1

Diets were formulated to have 30% inclusion rate of either rice or pea flours, while remaining closely isonitrogenous. GB, grain-based (rice) diet; Oligo, grain-based (rice) diet with 1% added raffinose, an oligosaccharide; GF, grain-free, pea-based diet.

### 2.3. Feeding trial

For 5–6 weeks prior to the start of the feeding trial, all dogs were fed a legume-free, veterinary-grade dental kibble as a husbandry diet (see Supplementary Table S1 for a list of ingredients of this diet). The dogs were then divided into three treatment groups and fed test diets twice daily over 5-week feeding periods in a blinded, randomized, crossover design. Body weights and body condition scores (BCS) using a 9-point scale were measured weekly. For several months prior to this feeding trial, individual daily portions were adjusted as needed to maintain BCS within ideal range (BCS 4–5), any uneaten food weighed to calculate actual food consumption and individual calorie requirement per day calculated from energy density (metabolizable energy or ME) of the husbandry diet according to the guaranteed analysis on the label. During the cross-over periods with our test diets, individual isoenergetic meal portions were calculated based on ME of test diet proximate analyses. All meal portions were kept to a consistent energy for each dog throughout the cross-over study, aside from one dog who required an 8% increase in portion size on the 3rd week of the first feeding period while on the Oligo diet due to unexplained weight loss and a drop in BCS below ideal. This dog regained her weight after 3 weeks and was able to maintain a stable weight within the ideal BCS at this increased portion size.

### 2.4. Nutrient and fiber analysis

Random 10–25 g subsamples from the test and husbandry diets were ground in the lab, then sent to Central Testing Laboratory Ltd. (Winnipeg, MB, Canada) for proximate and amino acid analysis according to AOAC standards as listed in Tables 2, 3. Dietary fiber content of all diets was assessed by Eurofins Scientific (Toronto ON, Canada) according to method AOAC 2011.25-M. Dietary fiber was differentiated into the categories of low molecular weight dietary fiber (LMWDF), insoluble high molecular weight dietary fiber (IHMWDF), and soluble high molecular weight dietary fiber (SHMWDF). LMWDF includes oligosaccharides, as well as other soluble and prebiotic fibers such as inulin, polydextrose and others. IHMWDF includes dietary fibers that do not dissolve in water, including cellulose and lignin, as well as resistant starch. SHMWDF consists of dietary fibers that dissolve in water, including hydrocolloids, gums, soluble pectin, and cereal ß-glucan. Individual amounts of the three main oligosaccharides found in legumes (raffinose, stachyose and verbascose) was also performed by Eurofins Scientific (29, 36).

**Table 2:** Proximate analysis of commercial husbandry diet and lab-formulated test diets.

Nutrient %DM (g/1000 kcal)	Husbandry		GB		Oligo		GF	
Metabolizable energy (kcal/kg)^1^	4,023		3,640		3,698		3,694	
Moisture^2^	8.1	(20)	10.7	(29)	12.9	(35)	10.6	(29)
Crude protein^3^	28.7	(71)	30.4	(84)	31.2	(84)	30.2	(82)
Crude fiber^4^	2.82	(7.0)	0.33	(0.9)	0.63	(1.7)	1.89	(5.1)
Fat^5^	13.2	(33)	10.6	(29)	11.2	(30)	10.9	(30)
Acid insoluble ash^6^	0.18	(0.5)	1.10	(3.0)	1.10	(3.0)	1.07	(3.0)
Non-fiber carbohydrates^7^	47.3	(118)	46.7	(128)	45.4	(123)	46.2	(125)
Total digestible nutrients^8^	81.2	(202)	79.7	(219)	79.8	(216)	79.3	(215)

% DM, percent dry matter; GB, grain-based (rice) diet; Oligo, grain-based (rice) diet with 1% added raffinose, an oligosaccharide; GF, grain-free, pea-based diet. ^1^Determined by Central Testing Laboratory Ltd. (Winnipeg, MB, Canada) using method AOAC 930.15. ^2^Determined by Central Testing Laboratory Ltd. (Winnipeg, MB, Canada) using method AOAC 930.03. ^3^Determined by Central Testing Laboratory Ltd. (Winnipeg, MB, Canada) using method by Ankom Technologies (2017) based on AOCS Ba 6a-05. ^4^Determined by Central Testing Laboratory Ltd. (Winnipeg, MB, Canada) using method AOAC A 5–04. ^5^Determined by Central Testing Laboratory Ltd. (Winnipeg, MB, Canada) using method AOAC 990.03. ^6^Determined by Central Testing Laboratory Ltd. (Winnipeg, MB, Canada) using method by J. Van Keulen and B.A. Young, AOAC 942.05. ^7^Determined by Central Testing Laboratory Ltd. (Winnipeg, MB, Canada) using equation Non-Fiber Carbohydrates (DM): 100 – [(% Dry Matter/100) + % Protein + %Fat + %Ash + %Crude Fiber]. ^8^Determined by Central Testing Laboratory Ltd. (Winnipeg, MB, Canada) using equation Total Digestible Nutrients (DM): 100 – [%Dry Matter/100) + %Ash + %Crude Fiber] – 8.

**Table 3:** Sulfur*-*containing amino acids cystine, methionine and taurine content of commercial husbandry diet and lab-formulated test diets.

Amino acid %DM (g/1000 kcal)	Husbandry		GB		Oligo		GF		AAFCO min requirements	
Cystine	0.46	(1.14)	0.30	(0.82)	0.38	(1.03)	0.26	(0.70)	N/A	
Methionine	0.54	(1.34)	0.33	(0.91)	0.43	(1.16)	0.24	(0.65)	0.33	(0.83)
Cystine + Methionine	1.00	(2.49)	0.63	(1.73)	0.81	(2.20)	0.50	(1.35)	0.65	(1.63)
Taurine	0.07	(0.17)	0.14	(0.38)	0.19	(0.51)	0.13	(0.35)	N/A	

% DM, percent dry matter; GB, grain-based (rice) diet; Oligo, grain-based (rice) diet with 1% added raffinose, an oligosaccharide; GF, grain-free, pea-based diet. Determined by Central Testing (Winnipeg, MB, Canada) using method AOAC 994.1.

### 2.5. Total tract apparent digestibility

Feces were collected during the final 2 days of each feeding period from each dog immediately after defecation (taken before feces fell to the ground or within a few seconds of landing on the ground) and stored at –20°C until used in further analyses. Samples were then pooled for a given individual, mixed and dried for 72 h at 65°C before being ground to a powdered consistency. Portions were sent to an external lab for proximate nutrient and amino acid analysis (Central Testing Laboratory Ltd., Winnipeg, MB, Canada). The Celite^®^ present in the feces was measured as acid insoluble ash (AIA) in the proximate analysis. This value was used to calculate total tract apparent digestibility using the formula (37):

$$NutrientDigestibility\;\left(\%\right)=\left[\;1-\left(\frac{\%\;\;nutrientinfeces\;x\;\;\%\;AIA\;infood}{\%\;\;nutrientinfood\;x\;\;\%\;AIA\;infeces}\right)\right]x\;100$$

### 2.6. Fecal bile acid analysis

A portion of the powdered, dried feces was used to measure total fecal bile acid content using a commercial assay kit (Total Bile Acid Assay Kit, Cell Biolabs Inc. Sand Diego, CA, USA). The assay measured bile acid content through a colorimetric enzyme driven reaction with 3ɑ-hydroxysteroid dehydrogenase, NADH and thio-NAD^+^. A second portion of feces was used to measure TCA content using a commercial assay kit (Taurocholic Acid (TCA) ELISA Kit, Abbexa LTD, Cambridge, UK). This ELISA used a competitive inhibition assay with a colorimetric detection.

### 2.7. Blood plasma analysis

Blood was collected in the morning on the final day of each test period after an overnight fast through jugular venipuncture. Samples of whole blood were collected into a heparinized and serum tubes, then sent to an external laboratory (Prairie Diagnostic Services, Saskatoon, SK, CA) to perform complete blood counts (CBC) and basic chemistry panels. Another portion of blood was collected in an EDTA tube and centrifuged at 2200 RPM for 10 min. Plasma was aliquoted and stored at-80°C until needed for further analyses. One set of plasma aliquots were sent to an external lab for measurement of the sulfur-containing amino acids cysteine/cystine, methionine and taurine (University of Victoria Proteomics Centre, Victoria, BC, CA) using Ultra-high Pressure Liquid Chromatography-Multiple Reaction Monitoring-Mass Spectrometry methodology. Other plasma aliquots were used in commercial kits for plasma canine NT-ProBNP and canine high-sensitivity cardiac specific troponin I (troponin) following manufacturer’s directions (Nordic BioSite Life Sciences, Täby, Sweden). Both of these latter kits were colourimetric-based immunoassays validated by the manufacturer for canine samples.

### 2.8. Echocardiographic assessment

All dogs were acclimated to the echocardiographic and blood pressure measurement procedures prior to beginning the feeding trial. On the 35th day of each feeding trial, echocardiograms were completed on the dogs while in fasted states according to previously described methods from this lab (13, 38). All measurements and imaging were performed by the primary researcher (EB). Training on ultrasound technique had been provided by an experienced veterinarian and greater than 200 h of experience performing ultrasonography was completed prior to the study. Blood pressure (BP) and heart rate were determined by the mean of 3 measurements taken from the base of the tail using a high-definition veterinary oscillometer (VET HDO® High Definition Oscillometry, Babenhausen, Germany). A Sonosite Edge II ultrasound machine (FUJIFILM Sonosite Inc., Toronto, ON, CA) was used to measure left ventricle chamber size and function during diastole and systole using Simpson’s rule for 2D ultrasonography in the left parasternal apical two-and four-chamber views. End-point measurements include ejection fraction (EF), cardiac output (CO), stroke volume (SV), left ventricle end diastolic volume (EDV), left ventricle end systolic volume (ESV) and peak mitral valve velocity during early diastole (Vmax E). Volumes were calculated using Simpson’s rule of disks. M-mode imaging was done through the right parasternal short-axis view at the level of the papillary muscles to assess left ventricular internal diameter during diastole (LVIDd) and systole (LVIDs). End-point measurements were taken as an average from at least two values per individual collected in separate cine loops during the exam. Values collected from echocardiographic assessment were normalized to the dog body weight for Simpson’s rule volumes, while M-mode obtained values for LVIDd and LVIDs measurements were standardized to different exponents of body weight according to guidelines suggested by Visser et al. (39).

### 2.9. Data handling and statistics

Data was analyzed using IBM SPSS 28 Statistics Software (IBM SPSS Statistics Software, Armonk, NY, USA). Data was tested for normality using the Kolomogorov–Smirnov (KS) test and homogeneity of variance using Levene’s test. When appropriate, data was log transformed to meet normality. If parametric assumptions were met, a repeated-measures, one-way ANOVA was performed, followed by Fisher’s least significant difference *post-hoc* test if significance was found (*p* ≤ 0.05). If normality was not met, analysis was done using Friedman’s test for ranked data.

## 3. Results

### 3.1. Diet proximate analysis

The three lab-formulated test diets contained similar amounts of metabolizable energy, crude protein, and crude fat per (Table 2). Calorie density fell between 3,640 kcal/kg to 3,698 kcal/kg among the test diets, while the husbandry diet contained higher energy content at 4,023 kcal/kg. Crude protein content ranged from 28.7–31.2%DM (71.3–84.4 g/1000 kcal) among all diets, all much higher than minimum protein requirements for adult dog maintenance of 18%DM (45 g/1000 kcal) as established by AAFCO (40). Crude fat content was highest in the husbandry diet at 13.2%DM (32.8 g/1000 kcal), while the formulated test diets contained similar crude fat content ranging from 10.6–11.2%DM (29.1–30.0 g/1000 kcal). Crude fiber ranged from 0.33–2.82 %DM (0.91–7.01 g/1000 kcal) among the diets, with the following rank order from lowest to highest: GB < Oligo < GF < husbandry diet.

#### 3.1.1. Fiber and amino acid content in diets

Total dietary fiber was found to be highest in the husbandry diet, and lowest in the GB and GF diet (Table 4). Quantity of low molecular weight dietary fiber (LMWDF) was highest in the GF diet, followed by the Oligo diet, while neither the husbandry nor GB diets had detectable levels of LMWDF (Table 4). While none of the test diets or the husbandry diets had detectable levels of the oligosaccharides, stachyose or verbascose, the 1% inclusion of raffinose in the Oligo diet resulted in similar raffinose content to the GF diet. In contrast, the GB diet and the husbandry diet contained no detectable levels of raffinose. Insoluble fiber was considerably higher in the husbandry diet compared to all test diets, while the GF diet had lower levels compared to both grain-containing test diets. The rank order of SHMWDF from lowest to highest was: GF = Oligo < husbandry diet < GB diet.

**Table 4:** Fiber content of the commercial husbandry diet and lab-formulated test diets.

Fiber parameter % w/w (g/1000 kcal)^1^	Husbandry	GB			Oligo		GF	
LMWDF	<0.6	(<1.6)	<0.6	(<1.8)	0.8	(2.5)	0.9	(2.7)
IHMWDF	6.6	(17.8)	2.9	(8.9)	3.2	(9.9)	2.6	(7.9)
SHMWDF	1.6	(4.3)	1.8	(5.5)	1.3	(4.0)	1.3	(3.9)
Total DF	8.2	(22.2)	4.7	(14.5)	5.3	(16.5)	4.8	(14.5)
Raffinose	<0.2	(<0.5)	<0.2	(<0.6)	0.8	(2.5)	0.8	(2.4)
Stachyose	<0.2	(<0.5)	<0.2	(<0.6)	<0.2	(<0.6)	<0.2	(<0.6)
Verbascose	<0.2	(<0.5)	<0.2	(<0.6)	<0.2	(<0.6)	<0.2	(<0.6)

% w/w, percent weight by weight; GB, grain-based (rice) diet; Oligo, grain-based (rice) diet with 1% added raffinose, an oligosaccharide; GF, grain-free, pea-based diet; DFLMWDF, low molecular weight dietary fiber; IHMWDF, insoluble high molecular weight dietary fiber; SHMWDF, soluble high molecular weight dietary fiber; DF, dietary fiber. ^1^Determined by Eurofins (Toronto ON, Canada) using method AOAC 2011.25-M.

The husbandry diet had a higher content of cysteine and methionine compared to all test diets, while containing lower taurine (Table 3). Among the test diets, the Oligo diet had the highest amount of all 3 sulfur-containing amino acids, followed by the GB diet. Analysis of amino acid content found that the methionine and methionine + cystine content of the GF diet fell below the AAFCO minimum requirements for adult dogs of 0.33 %DM (0.83 g/1000 kcal) methionine, and 0.65 %DM (1.63 g/1000 kcal) methionine + cystine. Methionine + cystine content of the GB diet also fell below minimum requirements when measured as %DM, but not if adjusted to g/1000 kcal.

### 3.2. Weight and condition

No significant differences in weight or body condition score were observed in dogs after being fed different test diets for 5 weeks (Table 5). Daily calorie provisions were similar among test diets, but greater in the husbandry diet. This was unintentional and due to a combination of overestimation of calorie amount in the formulations of the test diets, and the manufacturer’s reported calorie content being lower than what we later determined in proximate analyses after the feeding study was complete.

**Table 5:** Body weight, body condition score, daily portions, and daily calorie allotment (kcal/d) of dogs fed a commercial husbandry diet, grain-containing rice diets without (GB) or with (Oligo) the addition of the oligosaccharide raffinose, or a grain-free pea-based diet (GF).

	Husbandry	GB	Oligo	GF	*P-*value
Weight (kg)	9.26 ± 0.911	9.13 ± 0.858	9.16 ± 0.914	9.11 ± 0.874	0.476
BCS	4.7 ± 0.28	4.8 ± 0.164	4.5 ± 0.21	4.6 ± 0.18	0.613
Daily portion (g)	175 ± 19.8	180 ± 18.4	176 ± 18.7	179 ± 18.4	0.401
Kcal/d (kcal)	707 ± 79.6^a^	655 ± 66.9^b^	652 ± 69.3^b^	662 ± 68.0^b^	0.001

BCS, body condition score. *N* = 8 Data is shown as Mean ± SEM. Statistics with one-way repeated measures ANOVA. Different letters indicate significant differences in Fisher’s LSD *post hoc* analysis (*p* < 0.05).

### 3.3. Digestibility

Apparent total tract digestibility of crude protein, fat and non-fiber carbohydrate (NFC) was statistically similar across the test diets (Table 6). Digestibility could not be measured in the commercial dental husbandry diet since it lacked a non-digestible marker. Among the test diets, there was also no difference in digestibility of cysteine. However, methionine digestibility was lower in the GF diet compared to both GB and the Oligo diet. Taurine digestibility was also lower in the GF diet compared to the Oligo diet, but not the GB diet.

**Table 6:** Percent total tract apparent digestibility of test diets, including grain-containing rice diet without (GB) or with the addition of the oligosaccharide raffinose (Oligo), or a grain-free pea-based diet (GF).

	GB	Oligo	GF	*P-*value
Protein^1^	85.6 ± 2.54	84.4 ± 1.11	84.3 ± 0.45	0.849
Fat^1^	98.8 ± 0.15	98.4 ± 0.33	98.5 ± 0.24	0.537
Carbohydrate^1^	94.9 ± 1.14	95.0 ± 0.52	92.7 ± 0.60	0.142
Cysteine^2^	84.2 ± 3.32	85.7 ± 2.03	81.4 ± 1.69	0.404
Methionine^2^	93.5 ± 1.35^a^	94.5 ± 0.96^a^	89.6 ± 1.65^b^	0.006
Taurine^2^	84.9 ± 3.16^ab^	87.6 ± 2.14^a^	80.2 ± 3.16^b^	0.050
Total amino acids^2^	84.8 ± 2.82	86.6 ± 1.43	81.4 ± 1.33	0.203

*N* = 8 Data is shown as Mean ± SEM. Statistics with one-way repeated measures ANOVA. Different letters indicate significant differences in Fisher’s LSD *post hoc* analysis (*p* < 0.05). ^1^Determined by Central Testing (Winnipeg, MB, Canada) according to the methods listed in Table 2. ^2^Determined by Central Testing (Winnipeg, MB, Canada) using method AOAC 994.12.

### 3.4. Plasma amino acids

Plasma taurine levels were unchanged across all diets at the end of each 5-week feeding period (Table 7). In contrast, plasma methionine levels were significantly higher after dogs were fed the husbandry diet compared to either the Oligo diet or the GF diet, but intermediate after the GB diet. Cysteine/Cystine levels differed in dogs after feeding the different diets, with the significantly highest level observed in dogs after feeding the GB diet compared to after all other diets, while the husbandry diet had half this value that was significantly the lowest.

**Table 7:** Plasma levels of sulfur-containing amino acids of dogs fed grain-containing diets without (GB) or with the addition of the oligosaccharide raffinose (Oligo), or a grain-free pea-based diet (GF) over 5 week feeding periods.

Amino acid (μM)	Husbandry	GB	Oligo	GF	*P*-value
Taurine	74.3 ± 10.65	81.03 ± 9.70	74.29 ± 8.79	74.16 ± 7.31	0.521
Methionine	60.03 ± 3.12^a^	57.34 ± 4.05^ab^	54.38 ± 7.31^b^	53.24 ± 3.99^b^	0.023
Cysteine/Cystine	18.65 ± 1.35^a^	32.87 ± 3.23^b^	25.39 ± 9.70^c^	26.08 ± 2.53^c^	0.002

*N* = 8. Statistics with one-way repeated measures ANOVA. Different letters indicate significant differences in Fisher’s LSD *post hoc* analysis (*p* < 0.05).

### 3.5. Fecal bile acid and taurocholate analysis

Total fecal bile acid was significantly lower after dogs were fed the husbandry diet for 5-weeks compared to after the GB and GF diets, but there was no statistical difference among the test diets (Table 8). No statistically significant difference in TCA excretion or the ratio of this parameter to total bile acids across the any of the diets was observed.

**Table 8:** Bile acid content per gram feces, including both total bile acid (total BA) and taurocholic acid (TCA) quantity in dogs fed grain-containing diets without (GB) or with the addition of the oligosaccharide raffinose (Oligo), or a grain-free pea-based diet (GF) over 5 week feeding periods.

	Husbandry	GB	Oligo	GF	*P*-value
Total BA (μM/g)	45.6 ± 2.1^a^	53.1 ± 2.2^b^	49.7 ± 0.9^ab^	52.4 ± 0.7^b^	0.033^1^
TCA (nM/g)	80.8 ± 45.1	41.4 ± 27.9	10.6 ± 2.6	12.4 ± 3.6	0.075^2^
TCA to Total BA (%)	0.218 ± 0.140	0.073 ± 0.048	0.022 ± 0.007	0.024 ± 0.007	0.051^1^

*N* = 8. Data is shown as mean ± SEM. Analysis was done with ^1^one-way repeated measures ANOVA and ^2^Friedmans nonparametric test. Different letters indicate statistical differences using Fisher’s least significant difference *post-hoc* analysis (*p* < 0.05).

### 3.6. Blood and plasma analysis

The CBC showed no difference in white blood count among any of the diets after feeding them for 5-weeks (Table 9). Red blood cell count fell below the lower limit of the reference range after feeding the husbandry diet, which was significantly lower than what was seen with all other diets. Both hemoglobin and hematocrit were also found to be statistically lower after feeding dogs the husbandry diet compared to after any of the test diets. Mean corpuscular volume was higher while mean corpuscular hemoglobin and mean corpuscular hemoglobin concentration were both significantly lower after feeding the husbandry diet compared to after feeding the test diets. Red cell distribution width was not statistically different among any of the diets, and except for red blood cells, all values remained within the reference ranges.

**Table 9:** Complete blood count (CBC) and serum cholesterol of dogs fed grain-containing diets without (GB) or with (Oligo) the addition of the oligosaccharide raffinose, or a grain-free pea-based diet (GF) over 5 week feeding periods.

	Reference range	Husbandry	GB	Oligo	GF	*P*-value
WBC (10^9^/L)	4.9–15.4	6.3 ± 0.43	6.0 ± 0.50	5.7 ± 0.38	5.9 ± 0.49	0.567
RBC (10^12^/L)	5.80–8.50	5.67 ± 0.17^a^	6.21 ± 0.145^b^	6.13 ± 0.15^b^	6.15 ± 0.15^b^	<0.001
HGB (g/L)	133–197	135 ± 3.0^a^	150 ± 2.7^b^	148 ± 3.0^b^	149 ± 1.9^b^	<0.001
HCT (L/L)	0.390–0.560	0.405 ± 0.008^a^	0.434 ± 0.007^b^	0.429 ± 0.008^b^	0.429 ± 0.007^b^	0.019
MCV (fL)	62.0–72.0	71.6 ± 1.26^a^	70.1 ± 0.80^b^	70.0 ± 0.92^b^	70.0 ± 0.79^b^	0.012
MCH (pg)	21.0–25.0	23.9 ± 0.33^a^	24.1 ± 0.32^b^	24.3 ± 0.32^b^	24.2 ± 0.21^b^	0.026
MCHC (g/L)	330–360	333 ± 2.0^a^	344 ± 2.4^b^	347 ± 1.3^b^	346 ± 2.2^b^	<0.001
RDW (%)	11–14	12.7 ± 0.62	13.1 ± 0.26	12.8 ± 0.50	12.8 ± 0.36	0.080
Cholesterol (mmol/L)	2.70—5.94	5.04 ± 0.406^a^	4.27 ± 0.241^b^	4.14 ± 0.211^b^	3.96 ± 0.186^b^	<0.001

*N* = 8. Data is shown as mean ± SEM. Analysis was done with one-way repeated measures ANOVA. Different letters indicate statistical differences using Fisher’s least significant difference *post-hoc* analysis (*p* < 0.05). Reference range provided to show if values fall outside of normal limits and were for qualitative comparison only to experimental results. WBC, white blood cell; RBC, red blood cell count; HGB, hemoglobin; HCT, hematocrit; MCV, mean corpuscular volume; MCH, mean corpuscular hemoglobin; MCHC, mean corpuscular hemoglobin concentration; RDW, red cell distribution width.

Blood indicators of renal function, digestive enzymes and fasting blood glucose showed no statistical differences after dogs were fed diets and all values remained within the reference ranges (Supplementary Table S2). Indicators of hepatic function fell within the reference range aside from total protein, which ranged from 51 to 52 g/L, which is below the reference range, but with no statistical differences among the diets (Supplementary Table S3). Cholesterol levels remained within reference range, but were statistically higher in dogs after they were fed the husbandry diet compared to after the test diets. Total bilirubin significantly decreased, while alkaline phosphate increased in dogs after being fed the husbandry diet compared to after all test diets. Globulin levels in dogs after being fed the husbandry diet were significantly higher than after being fed the GF diet, while the GB and Oligo diet were intermediate and did not differ from any other. Albumin to globulin ratio was significantly lower in the dogs after being fed the husbandry diet compared to after the Oligo and GF diets.

In general, blood electrolyte levels all remained within the reference range across all diets, and no statistical differences were seen between in dogs after any of the diets (Supplementary Table S4). Exceptions were a significant reduction in bicarbonate and an increased anion gap in dogs after being fed the husbandry diet compared to other test diets, but both values remained well within normal reference range.

### 3.7. Plasma N-terminal pro-brain natriuretic peptide and cardiac-specific troponin I

**Figure 1:**
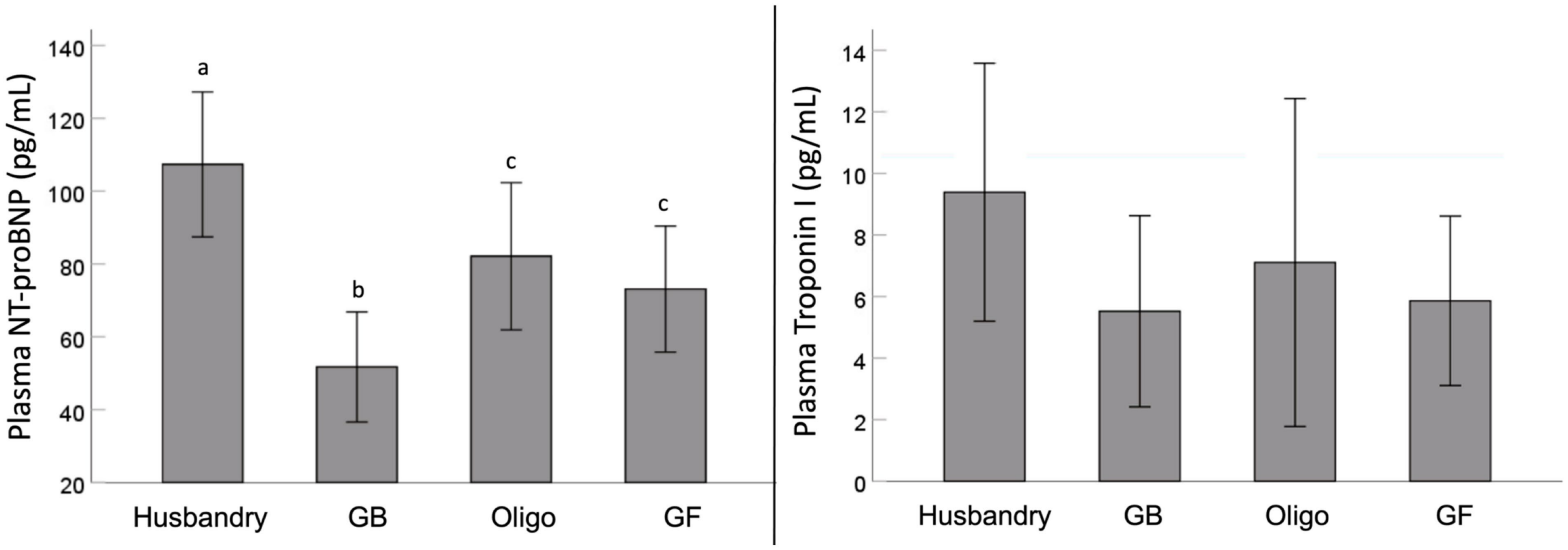
Plasma levels of N-terminal pro-brain natriuretic peptide (NT-proBNP) and high sensitivity cardiac specific troponin I (Troponin I) levels of dogs fed grain-containing diets without (GB) or with the addition of the oligosaccharide raffinose (Oligo), or a grain-free pea-based diet (GF) over 5 week feeding periods. Different letters indicate statistical differences using Fisher’s least significant differenes *post hoc* analysis.

We found significant differences in plasma NT-proBNP after feeding dogs the different diets (Figure 1). Specifically, after being fed the husbandry diet for 5 weeks, dogs had significantly higher NT-proBNP levels than after all other diets. In addition, NT-proBNP levels in dogs after being fed the Oligo and GF diets were intermediate to this, but statistically greater than after the GB diet. Although a similar trend was observed for mean plasma troponin levels, owing to significant variability after feeding each diet, no statistically significant differences were detected.

### 3.8. Echocardiography and cardiovascular health

Blood pressure and heart rate did not differ in dogs after being fed the different diets for 5-weeks, with all values falling within the reference range for a healthy adult dog (Table 10). Ejection fraction (EF) in dogs after feeding the husbandry diet was significantly lower than after both grain-containing test diets but did not differ from after the GF diet (Table 10). After the GF diet, dogs also had a significantly lower EF compared to after the Oligo diet, but not after the GB diet. End systolic volume (ESV) in dogs after feeding the husbandry diet was significantly larger than after both grain containing diets. No other significant differences in the other echocardiogram measurements were detected in dogs after feeding any of the diets.

**Table 10:** Parameters of cardiac function determined with echocardiography in dogs fed grain-containing diets without (GB) or with the addition of the oligosaccharide raffinose (Oligo), or a grain-free pea-based diet (GF) over 5 week feeding periods.

	Husbandry	GB	Oligo	GF	*P*-value
DBP (mmHg)	66 ± 2.5	69 ± 2.6	68 ± 3.2	70 ± 1.8	0.685
SBP (mmHg)	120 ± 5.3	135 ± 4.2	129 ± 5.4	127 ± 3.4	0.142
Heart rate (BPM)	75 ± 5.7	79 ± 4.2	74 ± 4.6	79 ± 2.9	0.750
EF (%)	62.0 ± 1.77^a^	67.4 ± 1.96^bc^	68.8 ± 2.15^c^	63.9 ± 2.65^ab^	0.044
CO (L/min/kg)	0.10 ± 0.008	0.11 ± 0.007	0.11 ± 0.008	0.11 ± 0.007	0.803
SV (ml/kg)	1.4 ± 0.12	1.4 ± 0.12	1.5 ± 0.12	1.4 ± 0.08	0.611
EDV (ml/kg)	2.24 ± 0.132	2.09 ± 0.142	2.22 ± 0.147	2.19 ± 0.077	0.447
ESV (ml/kg)	0.84 ± 0.036^a^	0.68 ± 0.045^b^	0.70 ± 0.067^b^	0.80 ± 0.054^ab^	0.047
LVIDd (cm/kg^0.299^)	1.30 ± 0.053	1.27 ± 0.051	1.26 ± 0.068	1.33 ± 0.055	0.533
LVIDs (cm/kg^0.387^)	0.75 ± 0.042	0.71 ± 0.033	0.71 ± 0.040	0.76 ± 0.036	0.148
Vmax E (cm/s)	66.3 ± 5.14	65.9 ± 2.64	62.0 ± 3.47	63.4 ± 3.00	0.633

*N* = 8. Data is shown as mean ± SEM. Analysis was done with one-way repeated measures ANOVA. Different letters indicate statistical differences using Fisher’s least significant difference *post hoc* analysis (*p* < 0.05). DBP, diastolic blood pressure; SBP, systolic blood pressure; BPM, beats per minute; EF, ejection fraction; CO, cardiac output; SV, stroke volume; EDV, end diastolic volume; ESV, end systolic volume; LVIDd, left ventricular internal diameter – diastolic; LVIDs, left ventricular internal diameter – systolic; Vmax E, early-wave maximum velocity through the mitral valve. EDV, ESV, SV, and CO utilized volume measurements taken from 2/4-chamber views and Simpson’s method of disks, while LVIDs and LVIDd values were taken from *M*-mode echocardiogram.

## 4. Discussion

### 4.1. Important outcomes

The outcomes of this study support there being a role of fiber in the link between atypical DCM and diet, but not in the way we hypothesized. Notably, we saw adverse changes in the markers of cardiac health and function among the diets after only 5-weeks of feeding the diets, which reversed with diet change. We saw an increase in NT-proBNP after feeding both the Oligo and the GF diets compared to after feeding the GB diet, but we found no corresponding differences in cardiac function as measured by echocardiography. However, the results after feeding the commercial, grain-containing husbandry diet were most striking. The husbandry diet (a commercial dental diet) led to the greatest increase in NT-proBNP and a coincident lower EF secondary to increased ESV compared to the grain containing diets, all consistent with early subclinical DCM. These changes were independent of plasma taurine status, as it did not differ among any of the diets and stayed within the reference range of 41–97 μM (21).

### 4.2. Diets

We succeeded in developing test diets with similar nutrient profiles while incorporating equal amounts of either rice or pea flour. Pea flour was chosen because it is the most common legume among the diets reported by the FDA and has recently been identified as the primary ingredient of concern in a foodomic analysis of the original DCM cases (6, 41). Our inclusion of 1% w/w raffinose in the Oligo diet correctly approximated the naturally occurring oligosaccharide of our GF diet. We also maintained similar protein levels near 30% for all the test diets. This not only meets the AAFCO minimum protein requirements of 18%, but is comparable to high protein content of many premium pet foods on the market (34, 42). Levels of methionine and combined cystine/methionine fell below the required minimums established by AAFCO in both our GF and GB diet when measured as %DM, although the GB diet did meet the requirements when presented as g/1000 kcal (34). These levels of methionine and combined cystine/methionine are comparable to previous studies with pea-based diets from our lab where diets were intentionally low in sulfur-containing amino acids to push dogs to more readily develop DCM-like changes (13, 43). However, the high overall crude protein and higher taurine content of the diets in the current study could lessen concerns about the low levels of precursor amino acids and mimic current premium diets where purified taurine is now commonly included.

A legume-free, grain-containing dental diet was chosen as a husbandry diet to support dog oral health and fed for 5–6 weeks pre-study. Crude protein and non-fiber carbohydrates content in the husbandry diet was similar to the test diets, but calorie density and fat content was slightly greater. Dental diets often include higher amount of certain dietary fibers to facilitate the removal of dental plaque through abrasion (44, 45). This is consistent with what we found in our husbandry diet, which listed both dried beet pulp and cellulose in the ingredient list, leading to considerably higher in IHMWDF compared to the test diets (Supplementary Table S1). The subclinical DCM-like cardiac changes observed after feeding the husbandry diet, which contained no measurable oligosaccharides, does not support our hypothesis that oligosaccharides are responsible for the development of nutrition related DCM.

### 4.3. Amino acid digestibility and plasma concentration

Due to the known link between taurine deficiency and some cases of DCM, we compared the effects of the diets on taurine and its precursor amino acids methionine and cysteine. The digestibility of the amino acids methionine and taurine were lower in the GF diet compared to the Oligo diet, agreeing with previous studies from this lab (43). This suggests that raffinose does not negatively impact the digestibility of sulfur containing amino acids, keeping in mind that results of apparent fecal digestibility can be misleading when the microbiome can act upon that nutrient (46). Reduced sulfur amino acid digestibility after feeding pea-based GF diets was attributed to increased total dietary fiber and amylose content in a previous study (43). We did not see this same relationship since the husbandry, GB and Oligo diets contained higher amounts of IHMWDF, and the GB contained higher SHMWDF compared to the GF. However, amylose content was not specifically assessed in this study so we cannot dismiss this as also being a factor.

There was no difference in dog plasma taurine levels among the diets, despite differences in digestibility, and lower taurine content in the husbandry diet. This aligns with what our lab has seen previously in both 7 day and 28 day feeding periods of pulse-based diets compared to grain-based diets in Beagles (13, 38, 43, 47). Effects of diet on plasma taurine in other studies were less consistent. With observational studies reporting both higher and lower taurine levels after feeding grain-free diets (16, 48). Breed differences appear to be at play, with one study finding that Beagle dogs are less susceptible to taurine depletion compared to larger mixed breed dogs (33). This study also suggested that plasma taurine is a less sensitive indicator of taurine status compared to skeletal muscle as a proxy for cardiac muscle, which may offer some explanation for conflicting results.

While all values remained within the reference range in the current study, plasma methionine levels were highest in the dogs after feeding the husbandry diet (49). The higher plasma methionine may be due to the higher dietary methionine in the husbandry diet. However, this explanation does not address the lower plasma cystine/cysteine levels in dogs after feeding the husbandry diet, which is unexpected given that this diet contained higher amounts of cysteine compared with the test diets. The lower plasma cysteine/cystine may be related to taurine availability since taurine stores are maintained in part by increased taurine synthesis through cysteine degradation in the liver (50, 51). The need for increased taurine synthesis may also be behind the lower plasma cystine/cysteine in dogs after feeding the Oligo diet compared to after the GB despite greater amount of dietary cystine in the Oligo diet. This would suggest that the additional oligosaccharide did lead to increased taurine loss.

### 4.4. Fecal bile acid

When examining whether oligosaccharides may cause taurine depletion through increased fecal bile acid losses, we found no differences in total bile acid losses after feeding our test diets regardless of oligosaccharide content. However, we did see a reduction in total bile acid excretion with the husbandry diet, which contained no detectable oligosaccharides but higher IHMWDF. These results agree with what we have previously seen in our lab, where increased amounts of IHMWDF led to reduced fecal bile acid excretion (13, 43). The diets associated with decreased bile acid excretion in these previous studies also contained higher levels of oligosaccharides, but the results of the current study do not support a role for oligosaccharides, or at the very least not raffinose, in mediating decreased total fecal bile acid excretion.

Although there were large relative differences in the excretion of the primary bile acid TCA in the feces, the values did not meet the criteria for statistical significance due to a high variability. The proportion of TCA to total bile acids (BA) also presented large differences in variability, and only slightly missed the threshold of statistical significance (Friedman’s test *p* = 0.051). The TCA/BA proportion was nearly 10x greater in the husbandry diet compared to the Oligo and GF diets. Since oligosaccharides are known to promote overgrowth of intestinal microbes, the lower TCA excretion in the Oligo and GF could be explained by greater deconjugation of bile acids by the microbiome (52, 53). Pezzali et al. (54) looked at bile acid excretion in dogs fed grain-based and grain-free diets, and similarly found no difference in total bile acid excretion but did observe a greater excretion of primary bile acid and percent primary bile acid in the higher-oligosaccharide, grain-free diet. The differences seen in bile acid composition in both studies supports the premise that the gut microbiome is acting upon bile acids differently with different dietary treatments. These shifts in the microbiome may be impacting taurine availability, which could explain the discrepancy in plasma cysteine/cystine levels seen between the Oligo and GB diet. Although it is established that the gut microbiota can impact taurine levels, the role of the microbiome in the development of nutrition-related DCM remains largely unexplored, and there is a growing need for more thorough research in this area (22, 33, 55).

### 4.5. Basic health and blood analysis

Throughout this study all the dogs remained healthy, without any progression to clinically significant DCM or heart failure despite changes consistent with early DCM after dogs were fed the husbandry dental diet. Although the husbandry diet provided greater calories daily than the test diets, weight and body condition score remained consistent throughout the study. The reduced red blood cells and other changes in hemoglobin parameters after dogs were fed the husbandry diet were consistent with a mild anemia, agreeing with a previous similar study by Bakke et al. that involved a feeding trial of a high legume diet and a retrospective analysis of health records of dogs diagnosed with DCM (56). Our lab has also seen lowered red blood cells after both 7 and 28-day feeding periods of feeding diets high in insoluble fiber (13, 47). Although IHMWDF was not measured directly in the 7-day feeding trial, crude fiber provides a relative approximation of insoluble fiber content, since it does not capture any soluble fiber or oligosaccharide present.

In the current study, we observed an increase in total cholesterol levels in dogs after feeding the husbandry diet compared to our test diets. This agrees with a previous study reporting dogs diagnosed with DCM compared to healthy controls have coincident elevations in cholesterol (10). This observed increase is likely related to bile acid excretion, since bile acids are produced by the conjugation of taurine with cholesterol, and we saw lower fecal bile acids after dogs were fed the husbandry diet. This is consistent with the understanding that insoluble fiber does not bind to bile acids as readily as soluble fiber, so is less likely to facilitate their excretion in stool (57). Lower fecal bile acid excretion suggests a higher reuptake of bile acids, and less cholesterol lost in the feces (28, 31, 58).

### 4.6. Cardiac biomarkers and echocardiography

One of the most noteworthy results in our study is the diet-related differences observed with plasma NT-proBNP which is stimulated by cardiac stretch and can be indicative of the severity of DCM (59, 60). The increases we saw in plasma NT-proBNP after feeding the husbandry diet were coincident with impaired systolic function (reduced EF and increased ESV), consistent with early changes associated with DCM. In contrast, NT-proBNP was also significant higher in dogs after feeding the GF and Oligo diets compared to the GB control diet, albeit to a lesser extent than after the husbandry diet and with no corresponding cardiac function changes. Circulating NT-proBNP is recognized as being one of the earliest indicators of cardiac dysfunction, and its levels may rise before any physical changes can be observed (10). However, any increase in a short feeding period of only 5-weeks in a relatively resistant breed such as the Beagle indicates the potential to develop into overt DCM if fed longer term or to more susceptible breeds (9, 10, 60).

These results align with previous studies where DCM-related changes in cardiac function have been observed with short-term diet change (8, 13, 14, 17, 19, 48). Two prospective feeding studies observed significant echocardiographic changes consistent with subclinical DCM after feeding a high pea diets (13, 14). In both studies peas were the primary protein source, and it is likely that the very high pea content (~60%) is behind the observed functional changes. In contrast, the current study only saw an increase in NT-proBNP after feeding our GF diet, which had pea content (30%) that more closely matched levels found in standard commercial diets (42). Also of note, Quilliam et al. (13) included a wrinkled pea variety in their diet that is known to have much higher amylose, but similar oligosaccharide content, whereas commercial pet food uses almost exclusively smooth-pea varieties.

The husbandry dental diet was selected to help manage periodontal disease that is common among Beagle dogs and deemed appropriate because it did not contain pulses or any other ingredients of concern (61, 62). The poor outcomes seen with the husbandry diet led us to reconsider the role of oligosaccharides in the pathogenesis of nutrition-related DCM and look more closely at other forms of fiber. Although we saw an increase in NT-proBNP after feeding the Oligo diet compared to after the GB diet, the impact of raffinose on cardiac health was minimal compared to the changes we observed after feeding the husbandry diet that was high in insoluble fiber. Since pulses are often higher in insoluble fiber compared to grain flours, our observations are still consistent with a high inclusion of pulses being behind the development of diet-related DCM (63). The mechanism underlying this possible link is still unclear, but the differences we saw in primary versus secondary bile acid excretion suggest the microbiome may be involved.

### 4.7. Limitations

We acknowledge that there are limitations in our study, particularly the small sample size of 8 Beagles and lack of washout period. To offset these factors, we conducted the study in a crossover design, which increased power and decreased variability. We also randomized treatment to counteract the latter limitation. The use of Beagles may also be seen as a limitation, given that they are not predisposed to developing DCM or taurine-deficiency, but several previous studies have now demonstrated diet-induced cardiac changes in this breed (13, 21). Supporting the utility of Beagles for nutritionally-mediated DCM studies, the current study also detected subclinical DCM changes, which was reversed with diet change.

A second limitation was the fact that the husbandry diet was not part of the randomized periods used for the other test diets. Moreover, proximate analyses performed after the pre-trial period where we fed the husbandry diet revealed that the energy density was much higher than the guaranteed analysis on the label for this commercial diet. This led to the dogs being fed significantly higher energy during the pre-trial period than intended. This husbandry diet also had higher sulfur amino acid levels, thus would have been predicted to protect cardiac health, but the opposite was observed.

Another limitation may have arisen from the inclusion of potato flour, which was originally listed as an ingredient of concern by the FDA (1). However a recent foodomic analysis concluded that potatoes were not significantly represented among diets associated with the initial nutrition-related DCM cases, and reaffirmed that peas, or possibly lentils, were the main ingredient(s) of concern (41). Potato flour was chosen to balance the diet formulations due to its relatively low fiber and protein content. We recognize however, that potato flour is not devoid of protein or dietary fiber. Thus, the potato flour may have impacted amino acid balance, as well as modulated the secondary effects of extrusion and resulting kibble density or gelatinization on nutrient availability (64).

## 5. Conclusion

As seen previously in our lab, this study demonstrated cardiac outcomes consistent with early subclinical DCM in a Beagle model, which was reversed with dietary changes (13, 38). We observed that raffinose present in the diet, either added in purified form or due to inclusion of peas, is sufficient, but not necessary to increase NT-proBNP. However, no adverse cardiac function changes were coincident with this blood parameter change with either of the oligosaccharide-containing diets. This could be due to there being no link between raffinose and DCM in dogs, or alternatively, that higher pea inclusion or a longer feeding period is needed to see adverse cardiac changes from oligosaccharides. Most noteworthy from this study is that the poorer outcomes were observed after feeding the commercial dental diet, which contained no legumes or detectable oligosaccharides, but was instead high in IHMWDF from the addition of cellulose and beet pulp. We propose that the higher content of insoluble dietary fiber present in the dental diet may be linked to these poorer outcomes, but further research looking more directly at this relationship versus oligosaccharides is needed. An exploration of the role of the microbiome is also warranted considering the differences noted in primary and total bile acid excretion.

## Data availability statement

The raw data supporting the conclusions of this article will be made available by the authors, without undue reservation.

## Ethics statement

The animal study was reviewed and approved by Animal Research Ethics Board of the University of Saskatchewan.

## Author contributions

EB and LW designed the study and drafted the article. EB formulated test diets, conducted the study, and performed the data analysis. All authors contributed to editing the article, as well as interpreting and verifying results.

## Funding

Funding was provided by the National Sciences and Engineering Research Council of Canada (NSERC), and the Western College of Veterinary Medicine’s Companion Animal Health Fund. In-kind support was provided by Horizon Manufacturing Inc. (Rosthern, SK Canada) in the form of some of the feed ingredients. This company played no role in the design, execution, analysis of data or manuscript preparation from this study.

## Acknowledgments

The authors thank the Canadian Feed Research Centre for their help in food production, and the Animal Care Unit staff for their work in caring for the research Beagles. Special thanks to Navoda Senanayake for her help in caring for and handling the Beagles throughout the study period.

## Conflict of interest

The authors declare that the research was conducted in the absence of any commercial or financial relationships that could be construed as a potential conflict of interest.

## Publisher’s note

All claims expressed in this article are solely those of the authors and do not necessarily represent those of their affiliated organizations, or those of the publisher, the editors and the reviewers. Any product that may be evaluated in this article, or claim that may be made by its manufacturer, is not guaranteed or endorsed by the publisher.

## Supplementary material

The Supplementary material for this article can be found online at: https://www.frontiersin.org/articles/10.3389/fvets.2023.1183301/full#supplementary-material
