# Supplementary material for: Oligosaccharides and diet-related dilated cardiomyopathy in beagles
Source: Front Vet Sci. 2023 Jul 24;10:1183301. doi: 10.3389/fvets.2023.1183301 (PMC10411538; doi:10.3389/fvets.2023.1183301)
Supplement: Supplementary file 1 [file Data_Sheet_1.docx]

**Supplemental Material**

**Table S1:** Ingredients of commercial dental husbandry diet provided to dogs at baseline (pre-study)

Chicken, chicken meal, brewers rice, ground yellow corn, ground wheat, corn gluten meal, animal fat preserved with mixed-tocopherols, dried beet pulp, brewers dried yeast, powdered cellulose, dried egg product, animal liver flavor, glycerin, salt, potassium chloride, calcium carbonate, phosphoric acid, tetra sodium pyrophosphate, mono and dicalcium phosphate, Vitamin E supplement, L-Lysine monohydrochloride, choline chloride, L-ascorbyl-2-polyphosphate, zinc sulfate, ferrous sulfate, manganese sulfate, niacin, potassium sorbate, vitamin A supplement, calcium pantothenate, thiamine mononitrate, copper sulfate, Vitamin B-12 supplement, riboflavin supplement, pyridoxine hydrochloride, garlic oil, folic acid, menadione sodium bisulfite complex, calcium iodate, vitamin D3 supplement, biotin, sodium selenite, C-2621

**Table S2:** Blood parameters of renal function, digestive enzymes and fasting blood glucose of dogs fed commercial husbandry diet, grain-containing diets without (GB) or with (Oligo) the addition of the oligosaccharide raffinose, or a grain-free pea-based diet (GF) over 5 week feeding periods

|  | Reference | Husbandry | GB | Oligo | GF | p value |
| --- | --- | --- | --- | --- | --- | --- |
| Urea (mmol/L) | 3.5 – 11.4 | 5.5 ± 0.24 | 5.6 ± 0.17 | 5.7 ± 0.19 | 5.7 ± 0.25 | 0.762 |
| Creatinine (µmol/L) | 41 – 121 | 60 ± 3.4 | 59 ± 3.2 | 61 ± 3.3 | 61 ± 3.6 | 0.795 |
| Amylase (U/L) | 343 – 1375 | 458 ± 41.7 | 540 ± 54.5 | 676 ± 125.8 | 506 ± 41.1 | 0.180 |
| Lipase (U/L) | 25 – 353 | 46 ± 6.6 | 61 ± 9.1 | 91 ± 26.2 | 54 ± 8.0 | 0.182 |
| Glucose (mmol/L) | 3.1 – 6.3 | 4.4 ± 0.16 | 4.4 ± 0.18 | 4.6 ± 0.14 | 4.4 ± 0.13 | 0.612 |

N=8. Statistics with One-Way Repeated Measures ANOVA. Different letters indicate significant differences in Fisher’s LSD post-hoc analysis (p<0.05)

**Table S3:** Blood parameters of hepatic function of dogs fed grain-containing diets without (GB) or with (Oligo) the addition of the oligosaccharide raffinose, or a grain-free pea-based diet (GF) over 5 week feeding periods

|  | Reference | Husbandry | GB | Oligo | GF | p value |
| --- | --- | --- | --- | --- | --- | --- |
| TB (µmol/L) | 1.0 – 4.0 | 0.9 ± 0.15^a^ | 1.3 ± 0.09^b^ | 1.1 ± 0.15^b^ | 1.2 ± 0.15^b^ | 0.023 |
| DB (µmol/L) | 0 – 2.0 | 0.5 ± 0.06 | 0.6 ± 0.04 | 0.6 ± 0.09 | 0.6 ± 0.06 | 0.196 |
| IB (µmol/L) | 0 – 2.5 | 0.4 ± 0.16 | 0.8 ± 0.08 | 0.6 ± 0.10 | 0.5 ± 0.10 | 0.090 |
| ALP (U/L) | 9 – 90 | 63 ± 14.5^a^ | 42 ± 6.5^b^ | 37 ± 4.7^b^ | 42 ± 6.2^b^ | 0.007 |
| GGT (U/L) | 0 – 8 | 3 ± 0.5 | 1 ± 0.8 | 3 ± 1.1 | 2 ± 0.8 | 0.469 |
| ALT (U/L) | 19 – 59 | 23 ± 1.1 | 22 ± 1.1 | 21 ± 1.2 | 24 ± 1.4 | 0.091 |
| GLDH (U/L)^†^ | 0 – 7 | 2.1 ± 0.30 | 2.9 ± 0.35 | 2.5 ± 0.27 | 2.5 ± 0.27 | 0.326 |
| CK (U/L) | 51 – 418 | 129 ± 18.3 | 132 ± 14.4 | 113 ± 7.4 | 128 ± 13.7 | 0.745 |
| TP (g/L) | 55 – 71 | 51 ± 1.7 | 52 ± 1.0 | 52 ± 1.4 | 51 ± 0.8 | 0.587 |
| Albumin (g/L) | 32 – 42 | 31 ± 1.7 | 33 ± 1.0 | 33 ± 1.4 | 33 ± 1.2 | 0.150 |
| Globulin (g/L) | 20-34 | 20 ± 0.6^a^ | 19 ± 0.5^ab^ | 19 ± 0.5^ab^ | 18 ± 0.7^b^ | 0.026 |
| A:G | 1:06 – 1:82 | 1.58 ± 0.11^a^ | 1.73 ± 0.08^ab^ | 1.79 ± 10.10^b^ | 1.84 ± 0.13^b^ | 0.019 |

N=8. Statistics with One-Way Repeated Measures ANOVA or Friedmans repeated-measures ANOVA on ranked data^†^. Different letters indicate significant differences in Fisher’s LSD post-hoc analysis (p<0.05).

TB = total bilirubin; DB – direct bilirubin; IB = indirect bilirubin; ALP: Alkaline phosphatase; GGT: Gamma-glutamyl transferase; ALT: Alanine aminotransferase; GLDH: Glutamate dehydrogenase; CK: Creatinine kinase; TP = total protein; A:G: Albumin to globulin ratio.

**Table S4:** Blood electrolytes of dogs fed a commercial husbandry diet, or grain-containing diets without (GB) or with (Oligo) the addition of the oligosaccharide raffinose, or a grain-free pea-based diet (GF) over 5 week feeding periods

|  | Reference | Husbandry | GB | Oligo | GF | p value |
| --- | --- | --- | --- | --- | --- | --- |
| Na (mmol/L) | 140 – 153 | 146 ± 0.4 | 147 ± 0.6 | 146 ± 0.4 | 147 ± 0.5 | 0.228 |
| K (mmol/L) | 3.8 – 5.6 | 4.5 ± 0.06 | 4.5 ± 0.09 | 4.4 ± 0.05 | 4.6 ± 0.08 | 0.432 |
| Cl (mmol/L) | 105 – 120 | 114 ± 0.5 | 113 ± 0.5 | 113 ± 0.4 | 114 ± 0.5 | 0.367 |
| HCO_3_^-^  (mmol/L) | 15 – 25 | 19 ± 0.5^a^ | 21 ± 0.2^b^ | 21± 0.3^b^ | 21 ± 0.3^b^ | <0.001 |
| Anion Gap (mmol/L) | 12 – 26 | 18 ± 0.5^a^ | 17 ± 0.6^b^ | 16 ± 0.3^b^ | 16 ± 0.4^b^ | 0.027 |
| Ca (mmol/L) | 1.91 – 3.03 | 2.44 ± 0.038 | 2.46 ± 0.032 | 2.45 ± 0.037 | 2.48 ± 0.030 | 0.366 |
| P (mmol/L) | 0.63 – 2.41 | 1.21 ± 0.048 | 1.16 ± 0.029 | 1.18 ± 0.041 | 1.27 ± 0.065 | 0.063 |
| Mg (mmol/L) | 0.70 – 1.16 | 0.79 ± 0.018 | 0.81 ± 0.017 | 0.80 ± 0.017 | 0.81 ± 0.017 | 0.649 |

N=8. Statistics with One-Way Repeated Measures ANOVA. Different letters indicate significant differences in Fisher’s LSD post-hoc analysis (p<0.05)

Na: sodium; K: potassium; Cl: Chloride; HCO3-: Bicarbonate; Ca: Calcium; P: Phosphorous; Mg: Magnesium
